# Antenatal magnesium sulphate for neuroprotection of preterm babies: data analysis of inequities in treatment in England 2014 to 2024

**DOI:** 10.1136/bmjoq-2026-004143

**Published:** 2026-09-07

**Authors:** Hannah B Edwards, Lauren J Scott, Cheryl McQuire, Isobel L Ward, David Odd, Frank de Vocht, Karen Luyt

**Affiliations:** 1 Population Health Sciences, University of Bristol Medical School, Bristol, UK; 2 NIHR ARC West, Bristol, UK; 3 Population Medicine, Cardiff University School of Medicine, Cardiff, UK; 4 Neonatology, NHS Wales Cardiff and Vale University Health Board, Cardiff, UK; 5 Translational Health Sciences, University of Bristol Medical School, Bristol, UK

**Keywords:** Healthcare quality improvement, Health Equity, Health services research, Maternal Health Services, Obstetrics and gynecology

## Abstract

**Objectives:**

To identify (a) Sociodemographic differences in treatment with antenatal magnesium sulphate (MgSO4) for fetal neuroprotection in preterm birth and (b) Whether any sociodemographic differences have reduced over time, in conjunction with implementation of a national quality improvement (QI) programme to improve use of MgSO4.

**Design:**

Retrospective cohort study using routinely collected data.

**Setting:**

NHS England maternity units from 2014 to 2024.

**Participants:**

Mothers with a preterm baby born from 24 to under 30 weeks’ gestation and admitted to an NHS neonatal unit.

**Outcomes:**

Receipt of antenatal MgSO4.

**Results:**

Before implementation of the National *PReCePT* (Preventing Cerebral Palsy in Pre-Term Labour) Programme (NPP) in 2018, mothers from the North of England had lower odds of MgSO4 treatment than mothers from the South of England (OR 0.60, 95% CI 0.49 to 0.74). Following implementation of the NPP, there was no longer evidence for regional differences in treatment (OR 0.87, 95% CI 0.70 to 1.08). Pre-NPP, mothers from more deprived areas had lower odds of treatment than women from more affluent areas (OR 0.87, 95% CI 0.80 to 0.95), but again, post-NPP, there was no longer evidence for difference in use by deprivation status (OR 1.02, 95% CI 0.92 to 1.14, p value for interaction=0.022). White British mothers may have had slightly lower odds of treatment than mothers of other ethnic groups (OR 0.93, 95% CI 0.87 to 1.00), and this potential difference did not appear to change from pre to post-NPP (p value for interaction=0.235). There was little evidence that maternal age affected the odds of treatment.

**Conclusions:**

Previous sociodemographic differences in receipt of antenatal MgSO4 treatment for preterm birth appear to have equalised in England. This follows, and could be due in part to England’s universal implementation of the National *PReCePT* QI programme.

**WHAT IS ALREADY KNOWN ON THIS TOPIC**

- A national quality improvement programme (*PReCePT*) has recently helped to improve uptake of antenatal magnesium sulphate (MgSO4), a neuroprotective treatment to protect against cerebral palsy in preterm births.
- There are persistent inequities in many aspects of maternity and perinatal care in England, and overall improvements in care do not imply improvements in equity.

**WHAT THIS STUDY ADDS**

- Before implementation of *PReCePT*, mothers from the North of England, from more socioeconomically deprived areas, or of white British ethnicity, all had lower odds of receiving antenatal MgSO4 treatment.
- Over time, and following implementation of *PReCePT*, sociodemographic variations in treatment appear to have reduced.

**HOW THIS STUDY MIGHT AFFECT RESEARCH, PRACTICE OR POLICY**

- Perinatal optimisation programmes should formally evaluate and aim to improve equity of care.

**Strengths and limitations of this study**

- Study uses standardised, routinely, prospectively collected healthcare data from all NHS neonatal units in England reporting on about 40 000 preterm births.
- Data covered a 10-year period (2014 to 2024) and the intervention was implemented in 2018, which gives sufficient time for reliable trends to be observed.
- Statistical modelling estimated the total effects of each risk factor separately, adjusted for measured confounders, and models were developed a priori using Directed Acyclic Graphs.
- The dataset only includes data on live-born infants (and their mothers) who were admitted to a neonatal unit.
- Sociodemographic differences do not necessarily imply inequity, and some of the variations in treatment observed here could be due to other factors.
- There are other individual characteristics also relevant to equity of care that were not measured in this data, and so could not be evaluated here.

## Introduction

 Persistent inequities in maternity and perinatal care and outcomes are well documented in the UK. The *Royal College of Obstetricians and Gynaecologists* National Maternity and Perinatal Audit (2021) reported ethnic and socioeconomic differences in caesarean birth, postpartum blood loss, small-for-gestational-age births, Apgar scores, neonatal admissions and breastfeeding rates.^1^ *MBRRACE-UK* Confidential Enquiries have consistently identified ethnic inequities in the care of women who experienced stillbirth, neonatal death or maternal death.^2^ The *National Neonatal Audit Programme* has highlighted geographical variation in antenatal preterm optimisation interventions, deferred cord clamping, neonatal mortality and key morbidities such as bronchopulmonary dysplasia and necrotising enterocolitis.^3^ The 2025 report from the National Child Mortality Database showed clear patterning of child deaths by ethnicity and deprivation, with perinatal or neonatal events as the leading cause of death.^4^ Recent UK cohort studies confirm continuing ethnic, socioeconomic and maternal age-related inequities in stillbirth, preterm birth, fetal growth restriction, deferred cord clamping, temperature, breastfeeding and neonatal mortality.^5–9^ Comparable patterns have been reported in other high- and upper-middle-income countries, although there are some differences in findings between nations (potentially due to differences in their populations, sociodemographic comparison groups and coding and healthcare systems).^10–15^

Reducing these inequities is a stated priority for the UK government, which has recently established a national maternity and neonatal taskforce and committed to an independent investigation of maternity and neonatal services.^16 17^ Baroness Amos’ interim report from this investigation has already flagged inequities in care as a key issue.^18^

One important metric of perinatal care is administration of the drug magnesium sulphate (MgSO₄), antenatally to women in preterm labour. This is an important treatment because there is very strong evidence that MgSO₄ reduces the risk of cerebral palsy (CP) in preterm infants, by around 30%.^19^ Preventing CP is of major importance given its lifelong effects on motor, cognitive and sensory function, and its substantial personal and economic costs.^20 21^ Avoidable cases of CP account for half of NHS litigation costs, with average payouts exceeding £10 million per case.^22^ MgSO₄ is inexpensive and highly cost-effective, generating an estimated £1 million in lifetime societal savings per case prevented.^23 24^ It is recommended by both the National Institute for Health and Care Excellence (NICE)^25^ and the World Health Organisation (WHO).^26^ UK guidelines recommend that MgSO₄ is offered to mothers going into preterm labour (or with a planned preterm birth) between 20 and <30 weeks’ gestation. They recommend that it is ‘considered’ where birth is anticipated between 30 and <34 weeks’ gestation, and ‘discussed with the mother’ where birth is anticipated between 23 and <24 weeks’ gestation.^19–25^

Uptake of antenatal MgSO₄ in the UK has improved following implementation of the National *PReCePT* (Preventing CP in Pre-Term labour) quality improvement (QI) programme in 2018.^27–29^ *PReCePT* was a QI intervention that helped inform and educate maternity staff about the importance of MgSO₄, and provided guidance and training for its administration. It provided maternity units with clinical guidance, training and educational resources, aide-memoires, posters and leaflets for the unit and for parents, and protected time for midwife champions to lead local delivery. It was found to be an effective and cost-effective intervention to improve MgSO₄ uptake.^27–29^ However, overall healthcare QIs can mask, or even worsen underlying inequities in care.^12 30–34^ It is uncommon for QI interventions to properly evaluate, let alone address inequities.^35–37^ Given the pervasive inequities across other aspects of maternity and perinatal care, similar patterns in uptake of antenatal MgSO₄ are plausible. They are also crucial to address given that inequity in preventative treatment here will perpetuate costly, lifelong health and social inequities.

### Aims

This study aimed to identify (a) Sociodemographic differences in treatment with antenatal MgSO₄ and (b) Whether any sociodemographic differences have reduced following implementation of a national QI programme (the National *PReCePT* Programme (NPP)) to improve use of MgSO₄. Maternal age, socioeconomic deprivation, ethnicity and geographical region were the key factors of interest. Our hypothesis was that MgSO4 administration had become more equitable following implementation of the NPP, such that any previous sociodemographic differences in treatment should have reduced.

## Methods

### Design

This was a retrospective cohort study using routinely collected healthcare data.

### Study setting and population

The study setting was England, January 2014 to December 2024. The population was mothers of babies born preterm between 24 weeks and zero days gestation (24^+0^) and 29 weeks and 6 days gestation (29^+6^) (inclusive). Data were extracted from the National Neonatal Research Database (NNRD), which holds data on all live-born preterm babies that were admitted to an NHS neonatal unit in England.^38^

### Outcomes and exposures

The outcome was receipt of MgSO4. Exposures of interest were maternal age (≥35 vs <35 years, as age 35 and over is the definition of a ‘geriatric’ pregnancy); area deprivation (Index of Multiple Deprivation (IMD) decile,^39^ coded as the five most vs the five least deprived deciles); ethnicity (white British vs all other groups, based on mother’s declared ethnicity from the NHS England standard codes for ethnic group. This was coded as a binary variable due to the relatively small sample sizes in each of the non-white British/‘minority’ groups); and region (North vs South of England, coded by regional neonatal Operational Delivery Network(ODN)). The time periods of interest (pre- vs post-NPP) were defined at the hospital level, as although the NPP was formally rolled out in May 2018, individual hospitals varied in their start dates (from April 2018 to October 2020). Further details of variable coding are described in the study protocol.^40^

Covariates considered for model inclusion were smoking history (any record vs none, as recorded at booking); single versus multiple birth; number of previous pregnancies (none vs one or more); fever in labour; receipt of antibiotics in labour; premature rupture of membranes; meconium stained liquor; antenatal steroids given; gestational age (GA; continuous completed weeks and days); onset of labour (spontaneous preterm labour or not); mode of delivery (vaginal vs caesarean). There is evidence that these factors can be both sociodemographically patterned and associated with receipt of care.^14 41–50^ Covariate selection was informed by Directed Acyclic Graphs (DAGs), developed a priori by the research team.^51–53^ Online supplemental appendices 1–3 present the DAGs and minimally sufficient adjustment sets for each exposure of interest. Further details are available in the study protocol.^40^

### Analysis

Categorical data are presented as numbers and percentages, and continuous data are presented as means and Standard Deviation (SD), or medians and Inter-Quartile Range (IQR) if not normally distributed. Logistic regression models with the outcome being receipt of MgSO4 were developed for each of the four sociodemographic factors separately (maternal age, deprivation, ethnicity and region), generating Odds Ratios (OR) for receiving MgSO4 associated with that sociodemographic factor. Variables in the unadjusted models included the factor of interest, NPP period, and interaction between sociodemographic factor and NPP period. This interaction term was included to identify changes in associations from pre- to post-NPP. Models were also run without the interaction term to obtain the overall associations.

An adjusted model was then developed for each sociodemographic factor separately to estimate the *total effects* of each sociodemographic factor on the outcome, with adjustment for potentially confounding covariates as indicated by the DAGs. These additionally adjusted for date (calendar month and year, to account for the underlying time trend), and clustering at the level of the neonatal unit, as mothers attending the same unit are likely to experience more similar care compared to mothers attending different units.

Due to antenatal MgSO4 also having a therapeutic role in the management of pregnancy hypertension and pre-eclampsia, models were re-run excluding mothers with pregnancy hypertension as a sensitivity analysis.

As a further supplementary analysis, we also developed adjusted models to estimate the *direct effects* of each sociodemographic factor on the outcome (the direct effects representing the component of total effects not mediated by indirect causal pathways, online supplemental appendices 1–4). All models were specified a priori based on DAGs (online supplemental appendices 1–3 and study protocol^40^).

### Patient and public involvement

Patients and the public were involved in the conduct, reporting, and dissemination of the wider programme of work that this study is part of; for example, two mothers with experience of preterm birth were on the programme Steering Committee.

## Results

### Population description

Mother and baby characteristics were largely comparable pre- and post-NPP (table 1). There were potential areas of difference in reported ethnicity (51.8% white British pre-NPP, 45.1% post-NPP); smoking history (17.3% pre-NPP, 14.1% post-NPP); meconium-stained liquor (5.2% pre-NPP, 1.9% post-NPP); receipt of antibiotics in labour (27.5% pre-NPP, 16.5% post-NPP); spontaneous preterm labour (57.2% pre-NPP, 48.3% post-NPP) and mode of delivery (55.2% caesarean section pre-NPP, 63.6% post-NPP). MgSO4 uptake improved from a mean 48.3% across the pre-NPP period to a mean 86.5% across the post-NPP period. There was good data completeness for most fields but a few with higher levels of missing data (ethnicity, smoking history, number of previous pregnancies, spontaneous preterm labour, and in the earliest part of the dataset, MgSO4 receipt.

**Table 1:** Characteristics of preterm births (24^+0^ to 29^+6^ weeks gestation) in England 2014–2024

	Pre National PReCePT Programme (2014–2018*)	Post National PReCePT Programme (2018–2024*)
Number of mothers	16 625	18 377
Number of babies	19 076	20 698
Maternal age (mean, SD)	30.5 (6.2)	31.2 (6.1)
Missing	102	120
Maternal ethnicity (n, %)		
White British	8607 (51.8%)	8292 (45.1%)
All other groups	5421 (32.6%)	6071 (33.0%)
Missing	2597 (15.6%)	4014 (21.8%)
Maternal Index of Multiple Deprivation (median, IQR)	4 (2 to 7)	4 (2 to 7)
Missing	301	266
Region		
North of England	8055 (48.5%)	8883 (48.3%)
South of England	8570 (51.5%)	9494 (51.7%)
Smoking history (n, %)		
Yes	2874 (17.3%)	2593 (14.1%)
No	11 761 (70.7%)	13 116 (71.4%)
Missing	1990 (12.0%)	2668 (14.5%)
Multiple birth (n, %)	2336 (14.1%)	2208 (12.0%)
Number of previous pregnancies (n, %)		
0	5516 (33.2%)	6292 (34.2%)
>0	9984 (60.1%)	10 386 (56.5%)
Missing	1125 (6.8%)	1699 (9.2%)
Fever in labour (n, %)	951 (5.7%)	705 (3.8%)
Received antibiotics in labour (n, %)	4564 (27.5%)	3026 (16.5%)
Premature rupture of membranes (n, %)	2699 (16.2%)	3095 (16.8%)
Meconium-stained liquor (n, %)	868 (5.2%)	345 (1.9%)
Gestational age (weeks, median, IQR)	27.9 (26.3 to 29.0)	27.7 (26.3 to 28.9)
Spontaneous preterm labour (n, %)		
Yes	9512 (57.2%)	8882 (48.3%)
No	6214 (37.4%)	8202 (44.6%)
Missing	899 (5.4%)	1293 (7.0%)
Mode of delivery (n, %)		
Vaginal	7451 (44.8%)	6697 (36.4%)
Caesarean	9174 (55.2%)	11 680 (63.6%)
Antenatal steroids given (n, %)		
Yes	15 313 (92.1%)	17 241 (93.8%)
No	1234 (7.4%)	1110 (6.0%)
Missing	78 (0.5%)	26 (0.1%)
Antenatal magnesium sulphate given (n, %)		
Yes	8022 (48.3%)	15 902 (86.5%)
No	5643 (33.9%)	2397 (13.0%)
Missing	2960 (17.8%)	78 (0.4%)

* May 2018 was the NPP national launch date, but individual hospitals varied in their start dates. Figures presented are indexed on individual hospital start dates.

### Maternal age

**Figure 1:**
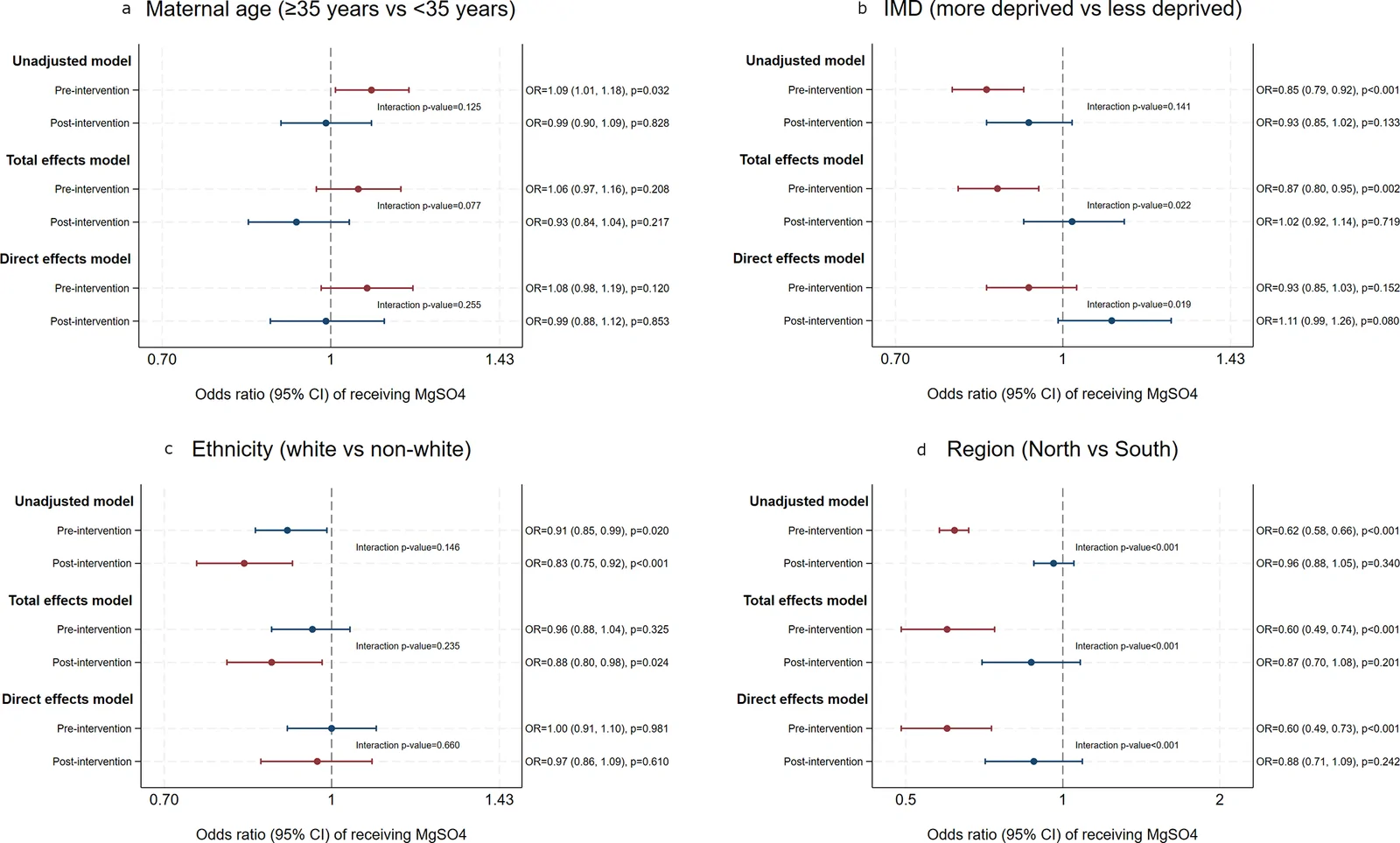
(**a**) The effect of maternal age on odds of antenatal MgSO_4_ treatment, pre- and post-National PReCePT Programme, (**b**) The effect of area deprivation on odds of antenatal MgSO_4_ treatment, pre and post National PReCePT Programme, (**c**) The effect of maternal ethnicity on odds of antenatal MgSO_4_ treatment, pre and post National PReCePT Programme and (**d**) The effect of region (North vs South of England) on odds of antenatal MgSO_4_ treatment, pre and post National PReCePT Programme. IMD, Index of Multiple Deprivation; MgSO_4_, magnesium sulphate.

There was little evidence that maternal age was associated with MgSO4 receipt (total effects adjusted OR (aOR) 1.01, 95% CI 0.94 to 1.08, p=0.839, with comparable results in the unadjusted model) (table 2). There was also little evidence for a change in this association from pre- to post-NPP, although coefficients suggested that older mothers may have had slightly higher odds of treatment pre-NPP that equalised post-NPP (table 2, figure 1a, online supplemental appendix 5).

**Table 2:** Total effects of sociodemographic factors on odds of receiving antenatal magnesium sulphate for preterm birth in England 2014–2024

	Unadjusted model* (OR, 95% CI)	Adjusted total effects model* (OR, 95% CI)
Maternal age (≥35 vs <35 years)		
Overall	1.05 (0.99 to 1.11), p=0.127	1.01 (0.94 to 1.08), p=0.839
P value for pre/post interaction	p=0.125	p=0.077
Pre intervention	1.09 (1.01 to 1.18), p=0.032	1.06 (0.97 to 1.16), p=0.208
Post intervention	0.99 (0.90 to 1.09), p=0.828	0.93 (0.84 to 1.04), p=0.217
Ethnicity (white vs non-white)		
Overall	0.88 (0.83 to 0.94), p<0.001	0.93 (0.87 to 1.00), p=0.038
P value for pre/post interaction	p=0.146	p=0.235
IMD (more vs less deprived)		
Overall	0.88 (0.83 to 0.93), p<0.001	0.93 (0.86 to 0.99), p=0.029
P value for pre/post interaction	p=0.141	p=0.022
Pre intervention	0.85 (0.79 to 0.92), p<0.001	0.87 (0.80 to 0.95), p=0.002
Post intervention	0.93 (0.85 to 1.02), p=0.133	1.02 (0.92 to 1.14), p=0.719
Region (North vs South)		
Overall	0.73 (0.69 to 0.77), p<0.001	0.70 (0.57 to 0.85), p<0.001
P value for pre/post interaction	p<0.001	p<0.001
Pre intervention	0.62 (0.58 to 0.66), p<0.001	0.60 (0.49 to 0.74), p<0.001
Post intervention	0.96 (0.88 to 1.05), p=0.340	0.87 (0.70 to 1.08), p=0.201

* See online supplemental appendix 1 for the list of covariates included in each individual model.

IMD, Index of Multiple Deprivation.

### Ethnicity

The unadjusted model showed that mothers of white British ethnicity had lower overall odds of treatment compared with mothers of other ethnicities, a difference that attenuated slightly after adjustment (aOR 0.93, 95% CI 0.87 to 1.00, p=0.038) (table 2). There was no evidence that this sociodemographic difference changed from pre- to post-NPP (table 2, figure 1b, online supplemental appendix 5).

### Area deprivation

The unadjusted model showed that mothers from more deprived areas had lower overall odds of MgSO4 treatment compared with mothers from less deprived areas, a difference that attenuated slightly after adjustment (aOR 0.93, 95% CI 0.86 to 0.99, p=0.029) (table 2). There was evidence for an improvement in this sociodemographic difference from pre- to post-NPP (interaction p=0.022): pre-NPP, mothers from more deprived areas had lower odds of treatment (aOR 0.87, 95% CI 0.80 to 0.95, p=0.002); post-NPP, there was no longer evidence that deprivation level affected odds of treatment (OR 1.02, 95% CI 0.92 to 1.14, p=0.719) (table 2, figure 1c, online supplemental appendix 5).

### Region

There was strong evidence for a significant regional difference in MgSO4 treatment: pre-NPP, mothers in the North of England had around 40% lower odds of treatment compared with mothers in the South of England (total effects aOR 0.60, 95% CI 0.49 to 0.74, p<0.001) (table 2). Post-NPP, there was no longer evidence that region affected odds of treatment (total effects aOR 0.87, 95% CI 0.70 to 1.08, p=0.201) (table 2, figure 1d, online supplemental appendix 5).

### Supplementary analyses

Statistical models adjusted for the *direct effects* of each sociodemographic factor (ie, excluding effects via indirect causal pathways) indicated that there was little evidence for a direct effect of maternal age, ethnicity or deprivation on odds of treatment. There did appear to be a direct effect of region, with estimates very comparable to those found in the total effects model (online supplemental appendix 4).

### Sensitivity analyses

Excluding mothers with a record of pregnancy hypertension produced results compatible with the main analyses (online supplemental appendices 6 and 7).

## Discussion

### Key findings

By far the strongest observed sociodemographic difference in antenatal MgSO4 treatment was between mothers in the North versus South of England: mothers in the North were nearly half as likely to receive antenatal MgSO4 compared with mothers in the South, prior to NPP implementation. This striking regional difference appeared to improve post-NPP. Mothers from more deprived areas were less likely to receive treatment compared with mothers from more affluent areas prior to NPP implementation. This difference also appeared to improve post-NPP. White British mothers were slightly less likely to receive MgSO4 treatment compared with mothers of other ethnicities (including white mothers of non-British ethnicity), and this difference appeared consistent throughout the study period. There was little evidence that maternal age affected odds of MgSO4 treatment, although estimates suggested that pre-NPP, older mothers may have had slightly higher odds of treatment.

### Comparison with other literature

The stark evidence for a historical, high-magnitude difference in treatment by North/South of England is consistent with other evidence on broader health inequities by the “North/South divide”.^3 54^ Inequity in protective antenatal care for preterm babies is particularly significant, as it drives (and may compound) a lifetime of health and economic burden for affected individuals, families and society.^55–57^ The difference observed here persisted through statistical adjustment for known key confounding factors including deprivation, ethnicity and smoking status, and other differences in population characteristics between the two periods. Although it is likely that there are other unmeasured differences not captured here (a key one being patient behaviour), it is reassuring that since implementation of the NPP, there is no longer evidence of this regional difference.

Deprivation is well-known to have adverse effects on receipt of healthcare, and is believed to act through multiple entrenched mechanisms and at multiple levels.^58^ Our findings that deprivation was associated with lower odds of MgSO4 treatment are consistent with this wider picture. How deprivation would drive differences in treatment in this context are not clear. The supplementary analysis found little evidence for a *direct* effect of deprivation on odds of MgSO4 treatment, indicating that this association is driven by indirect pathways. Further exploration of this would be valuable, but again it is reassuring that since implementation of the NPP, there is no longer evidence of this difference.

The slight disadvantage experienced by White British mothers in receipt of MgSO4 treatment is interesting, and counter to our hypothesis, as the direction of effect is opposite to that commonly found in other aspects of maternity and perinatal care. Commonly, black and ethnic minority groups are at a disadvantage in both receipt of care and health outcomes.^1 5–7 59^ However, comparable results of disadvantage to White British mothers in some aspects of maternity care have been found in the recent *MBRRACE* reports.^2^ Again, the supplementary analysis found little evidence for a *direct* effect of ethnicity on odds of MgSO4 treatment, indicating that this association is driven by indirect pathways (including, plausibly, factors not measured here). Further exploration of the relevant individual characteristics driving this association would be valuable. In particular, although difficult to measure, it would be interesting to further explore and untangle the relative contributions of cultural factors, race and nationality.

*Prima facie* it could be seen as surprising that both white British ethnicity and deprivation appear to have historically been associated with lower odds of treatment, given that on average in the UK, deprivation disproportionately affects ethnic minority rather than white British populations. However, there are two reasons that this should not be a significant concern: Firstly because it is plausible that this overall trend is not true in all sub-groups: the population studied here, mothers experiencing preterm birth, with babies surviving to neonatal admission, could plausibly overrepresent white British women of lower socio-economic status, meaning that the overall UK trend is not true for this specific population. Secondly, our analyses minimised potential confounding between ethnicity and deprivation by including adjustment for them in the models (as indicated by the DAGs).

Regarding the potential effects of maternal age, a registry study in Australia and New Zealand found that older mothers are slightly more likely to receive MgSO4,^14^ which is consistent with our estimates, although our results showed no strong statistical evidence for this association. The background secular trend, both in the UK and internationally, is of gradually increasing use of MgSO4.^60^ Our previous evaluations found evidence that the NPP contributed to the observed improvements in England.^27 28^ Although overall improvement does not necessarily have any implications for equity, it is plausible that the NPP, as a universal intervention, did have a role in reducing the sociodemographic differences in treatment observed here. Mothers who were less likely to be treated pre-NPP may have been the ones who benefitted most from the intervention. This fits with the broader pattern of where there is a ceiling effect on uptake, groups with a lower starting uptake have the most room for improvement.^61^ It is possible that other secular trends, for example, increasing government focus on health equity more broadly, have also contributed to the apparent improvements in equity (or at least reductions in sociodemographic differences) over time that were observed here. In our view the sociodemographic equalising appears to coincide with implementation of the NPP (as can be seen in online supplemental appendix 5), but we encourage readers to draw their own conclusions.

### Strengths and limitations

Key strengths of this study are that it uses standardised, routinely, prospectively collected healthcare data from all NHS neonatal units in England. This minimises biases in the data such as selection bias, observer bias and recall bias. Findings are generalisable across the country. Data covered a 10-year period of 2014 to 2024 and the NPP intervention was implemented in 2018, which gives sufficient time for reliable trends to be observed. Statistical modelling estimated the total effects of each sociodemographic factor, adjusted for measured potential confounders, and as supplementary analyses we additionally estimated the direct effects of each of these key sociodemographic factors. All models were developed a priori using DAGs, to clarify assumptions and guide design of the statistical models to minimise confounding and best estimate causal effects. Results were robust to sensitivity analyses.

Observed differences in receipt of a treatment do not always indicate inequity in care, and it is possible that some of the differences observed here are due in part to clinical, behavioural or even organisational factors. In some cases it could just reflect that different groups have different levels of need. However in this case, all mothers in this study are eligible for treatment, so are considered to have equal need. Medical contra-indication is rare and unlikely to be sociodemographically patterned. This suggests that the observed differences may reflect a true inequity in care. Differences in treatment could also be due to differences in patient behaviour – for example, if certain patient groups tend to arrive later at hospital, so have a smaller window of opportunity for treatment. Arguably such cases are not ‘inequity in care’, or at least not inequity at the point of care, but just variations in patient behaviour that lead to differential treatment rates. Further exploration of upstream patient behaviour would be valuable here to better understand the causal pathways leading to differences in treatment. However, again it could be argued that all of these mothers should be offered treatment, and if some groups are under-treated, that is arguably an inequity to be addressed, regardless of whether the reason applies at the point of care or further upstream.

A connected limitation is that we did not have data on all possible covariates of interest, so residual confounding is possible. For example maternal BMI, patterns and details of previous birth history (including assisted conception and stillbirths), how close the mother lives to their maternity unit, how early or late different groups of mothers tend to present at hospital, imminence of delivery, trajectory of labour, and timing of delivery could all potentially vary by patient group and affect receipt of MgSO4. Similarly, there are other individual characteristics that as well as being potential confounders, could also be considered important dimensions of equity themselves, for example, race, language, sexual orientation, disability status, chronic illness status, marital status, education, health literacy and urban/rural living^62^ – that were not measured in this data and so could not be evaluated here. If possible, it would be valuable for future work to include data on these other variables to help understand the pathways and reasons for variation in treatment.

It is possible that changes in population characteristics over time could be explaining some of the differences observed here. The post-NPP population tended to have slightly fewer smokers, slightly fewer cases of fever in labour or receiving antibiotics, fewer cases of meconium-stained liquor, more cases of spontaneous onset of labour and more caesarean sections. It is plausible that some of these factors are also associated with likelihood of treatment: for example, non-smokers may be more likely to be present and available to treat; and fewer ‘complications’ such as fever and meconium-stained liquor could also mean a bigger window of opportunity to treat between presentation and delivery. We tried to address this by adjusting for these factors wherever indicated in the DAGs, but residual confounding is possible, and of course there may be alternative views on the details of some of the relationships modelled in our DAGs.

Another limitation is that the neonatal dataset only includes data on live-born infants who were admitted to a neonatal unit, and their mothers. This is a sub-group of an underlying population of mothers and infants who may also have been treated with MgSO4 for expected preterm birth, but who either did not go on to have a preterm birth or where the infant died prior to NICU admission. It is possible that this selected population has a different distribution of sociodemographic characteristics to the underlying population, and that the odds of treatment for those selected into the dataset is different from that in the underlying population they represent. If possible, it would be a valuable future research study to replicate this work on the wider underlying population.

Although for most fields data completeness was high, two with potentially relevant missing data were maternal ethnicity (15–20% missing) and MgSO4 receipt (17% missing during the pre-NPP period). Ethnicity data is often not well recorded in routine data, and the amount missing here is at the lower end of nationally reported statistics (a 2025 NHS England report found 18%–32% missing ethnicity data across national records^63^). It is possible that this missing data could be biasing our findings, if the ethnicity-MgSO4 receipt association is systematically different in those with missing data compared with those with complete data. The impact of missing ethnicity data in this dataset has been investigated recently, and reassuringly was found to have minimal impact on estimates of inequalities.^9^

More granular detail on individual-level deprivation factors may be useful in future work. It is possible that factors not captured by the area-level IMD (or captured, but different at the individual vs area-level) could also drive differences in receipt of healthcare. Similarly, more granular detail in the differences in treatment between the different minority ethnic groups would be valuable, as it is possible that combining them – as was indicated in this study to give sufficient power – may conceal variation in sub-group treatment rates.^12^

For the minority of eligible mothers not receiving antenatal MgSO4, further exploration of the reasons why they did not – and whether or not this varies by sociodemographic factors – would be valuable. Previous research on this population indicates that the overwhelming reported reason for not receiving treatment (>75% of cases) is ‘imminent delivery’, with smaller proportions reported as ‘not offered’ (11%), ‘not appropriate’ (5%) and very rarely ‘contraindicated’ or ‘declined’.^27^ In this study the sample size would not have been sufficient to power further analysis of this by sociodemographic sub-groups, but if numbers allowed in larger, international datasets (eg, the Vermont Oxford Network dataset^64^), further analysis on this question would be useful.

## Conclusions

Receipt of MgSO4 was patterned by deprivation, ethnicity and possibly maternal age, as well as showing wide regional variation. Over time and in conjunction with the roll-out of *PReCePT*, a national QI programme that increased overall use of MgSO4, these sociodemographic differences in care appear to have reduced (although White British mothers may still have slightly lower odds of treatment than mothers of other ethnicities). This study illustrates that a universal, nationally implemented QI programme may have a role in improving equity of care as well as overall treatment uptake. It also highlights the importance of evaluating trends in equity and sociodemographic variations more broadly, in other metrics of maternal and perinatal care.

## Supplementary material

10.1136/bmjoq-2026-004143 online supplemental appendix 1

## Data Availability

Data may be obtained from a third party and are not publicly available.
