# Supplementary material for: Antenatal magnesium sulphate for neuroprotection of preterm babies: data analysis of inequities in treatment in England 2014 to 2024
Source: BMJ Open Qual. 2026 Sep 7;15(3):e004143. doi: 10.1136/bmjoq-2026-004143 (PMC13561023; doi:10.1136/bmjoq-2026-004143)
Supplement: Supplementary file 1 [file bmjoq-15-3-s001.pdf]

Supplementary materials

List of abbreviations

ANS	Antenatal steroids
GA	Gestational age
IMD	Index of Multiple Deprivation (a measure of area deprivation)
IVF	In-vitro fertilisation
MgSO4	Magnesium sulphate
Multiple	Multiple birth (twin, triplet, etc)
NPP	National PReCePT Programme
PROM	Premature rupture of membranes

Appendix 1: Adjustment sets for statistical models

**Exposure of interest**	**Outcome**	**Main model 1: Unadjusted**	**Main model 2: TOTAL EFFECTS**	**Supplementary model 3: DIRECT EFFECTS***
Maternal age (≥35 vs <35)	$MgSO_4$	Pre/Post NPP<br><br>Interaction between exposure and Pre/Post NPP	*Model 1 +*<br>IMD<br>Ethnicity<br>Region<br><br>+date<br>+clustering on unit	*Model 2 +*<br>GA<br>Multiple<br>Previous pregnancies<br>Smoking
Ethnicity (white British vs other)	$MgSO_4$	Pre/Post NPP<br><br>Interaction between exposure and Pre/Post NPP	*Model 1 +*<br>No others<br>*("No open biasing paths. No adjustment is necessary to estimate the total effect of ethnicity on MgSO4")*<br><br>+date<br>+clustering on unit	*Model 2 +*<br>IMD<br>Maternal age<br>Previous pregnancies<br>Region<br>Smoking
IMD (high vs low deprivation)	$MgSO_4$	Pre/Post NPP<br><br>Interaction between exposure and Pre/Post NPP	*Model 1 +*<br>Ethnicity<br>Region<br><br>+date<br>+clustering on unit	*Model 2 +*<br>GA<br>Delivery mode<br>Fever in labour<br>Maternal age<br>Multiple<br>Onset of labour<br>Previous pregnancies<br>PROM<br>Smoking
Region (North vs South)	$MgSO_4$	Pre/Post NPP<br><br>Interaction between exposure and Pre/Post NPP	*Model 1 +*<br>Ethnicity<br><br>+date<br>+clustering on unit	*Model 2 +*<br>GA<br>IMD<br>Delivery mode<br>Fever in labour<br>Maternal age<br>Multiple<br>Onset of labour<br>Previous pregnancies<br>PROM<br>Smoking

* The main analysis was to estimate the *total effects* of each sociodemographic factor on odds of receiving $MgSO_4$. As a supplementary analysis, we also developed adjusted models to estimate the *direct effects* of each sociodemographic factor on the outcome. *Direct effects* represent the component of total effects not mediated by indirect causal pathways(1). For example, the effect of maternal age on receiving $MgSO_4$ is likely to act via both direct and indirect pathways; older mothers may have higher odds of treatment as they fall into a higher-risk pregnancy category (a *direct effect* of age), while they may also be more likely to be non-smokers, with higher odds of treatment (an *indirect effect*). In this case, smoking status would be adjusted for in the model that estimates only the *direct effect* of maternal age, but would not be adjusted for in the *total effects* model, which would consider effects across all direct (biological age) and indirect (exposure to smoking) pathways.

1. Tennant PWG, Murray EJ, Arnold KF, Berrie L, Fox MP, Gadd SC, et al. Use of directed acyclic graphs (DAGs) to identify confounders in applied health research: review and recommendations. International Journal of Epidemiology. 2020;50(2):620-32.

Appendix 2: Directed Acyclic Graphs for statistical models

(a) Maternal age *total effects* model

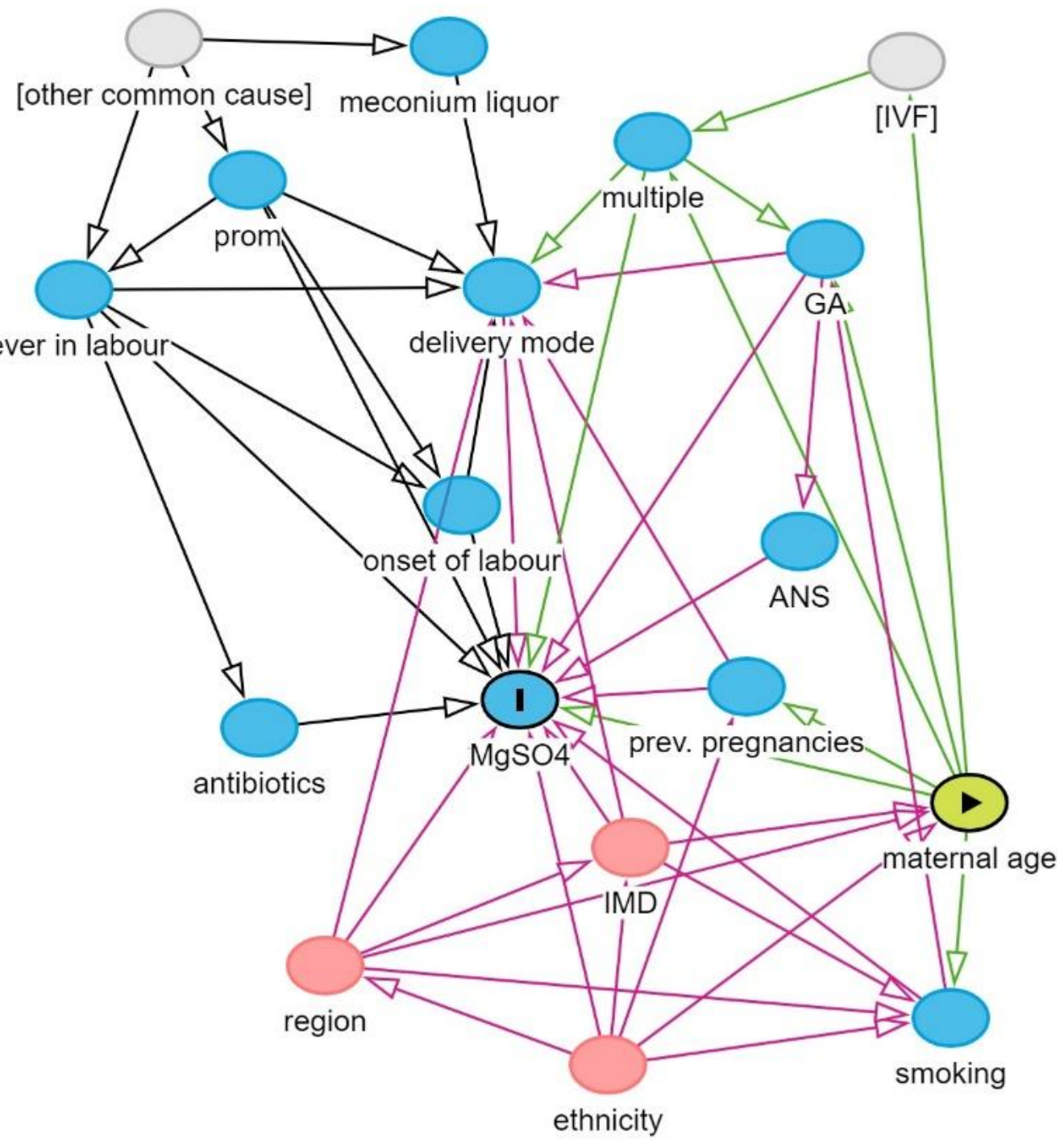

(b) Maternal age *direct effects* model

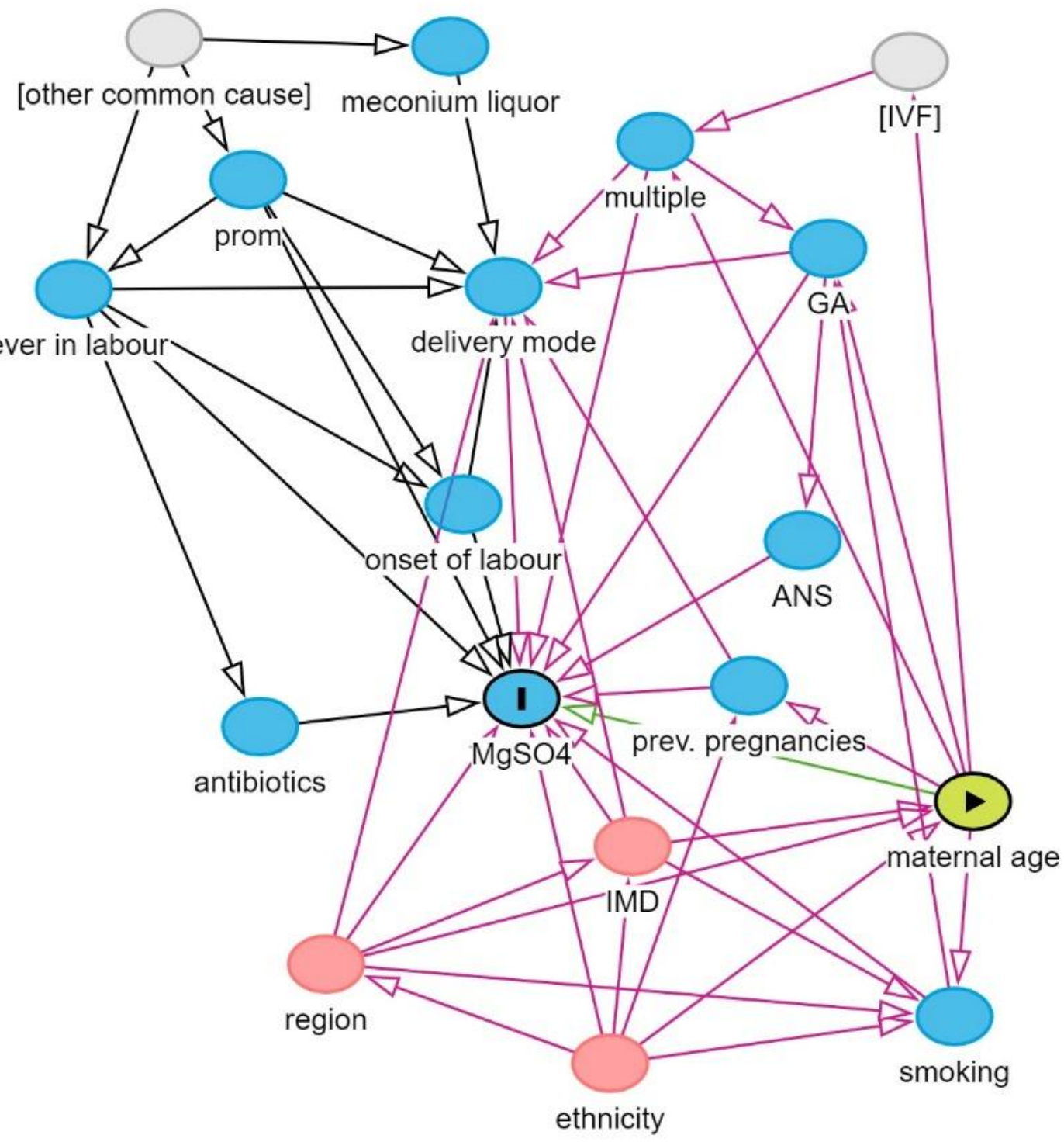

(c) Maternal IMD *total effects* model

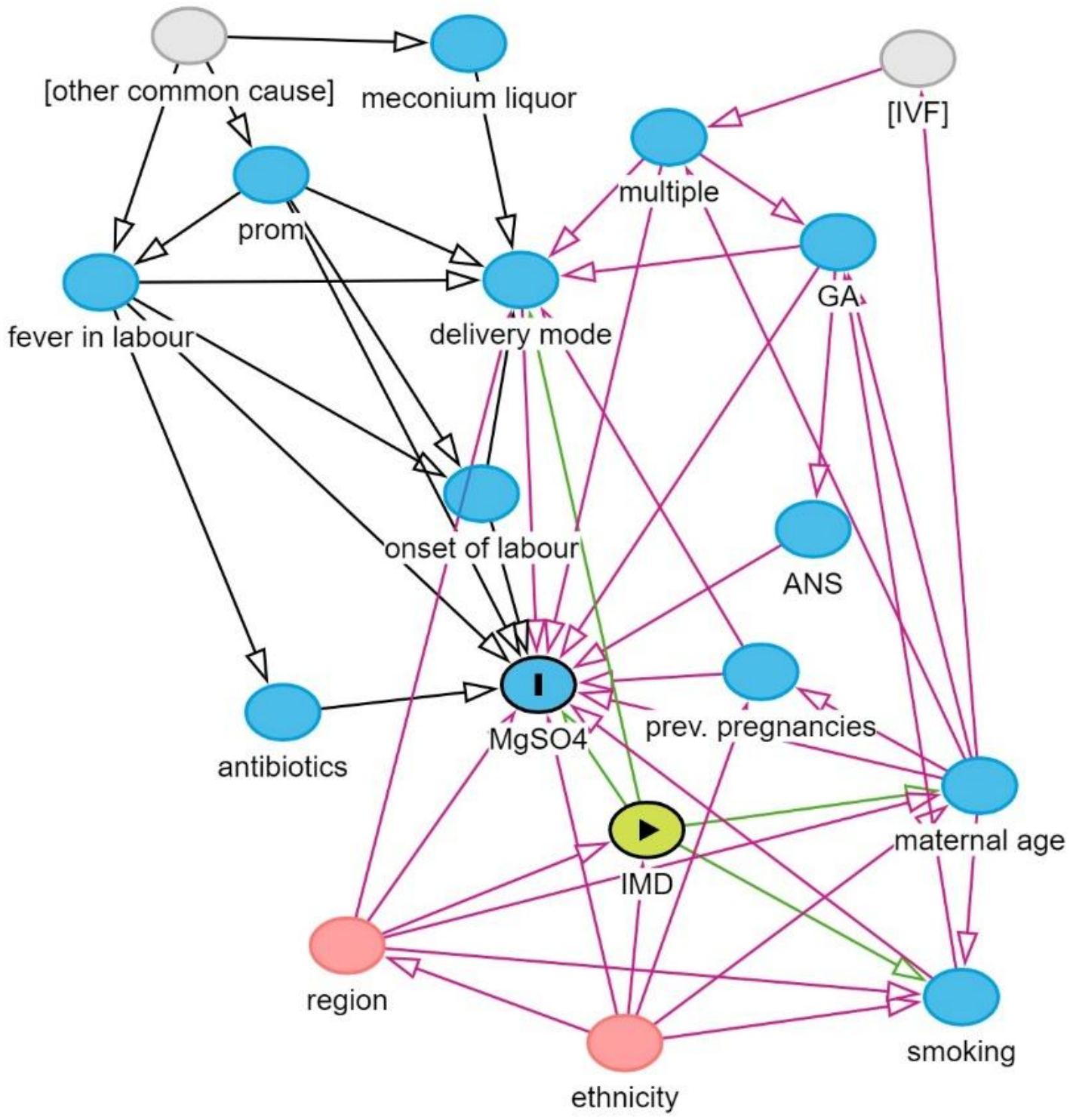

(d) Maternal IMD *direct effects* model

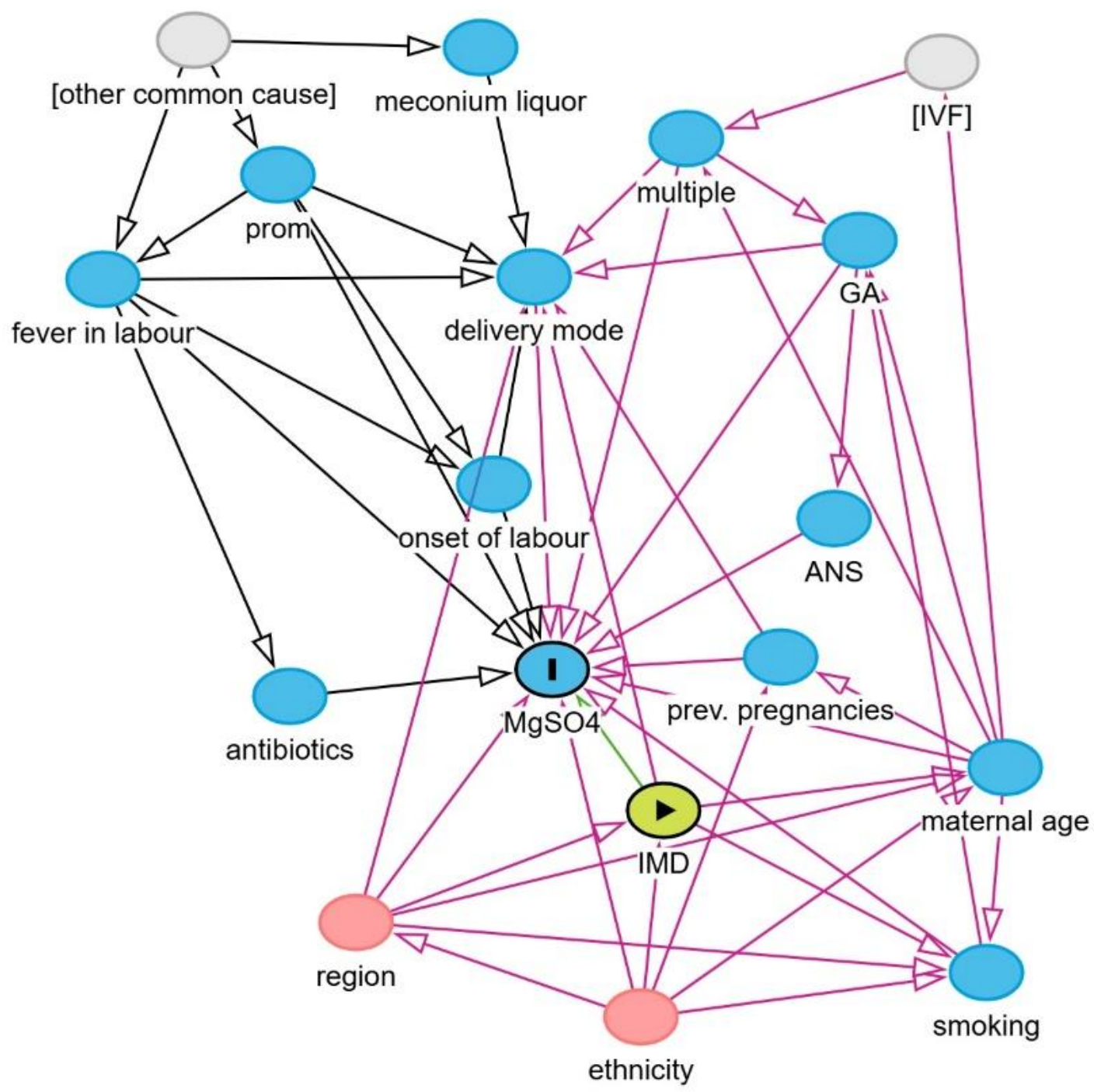

(e) Maternal ethnicity *total effects* model

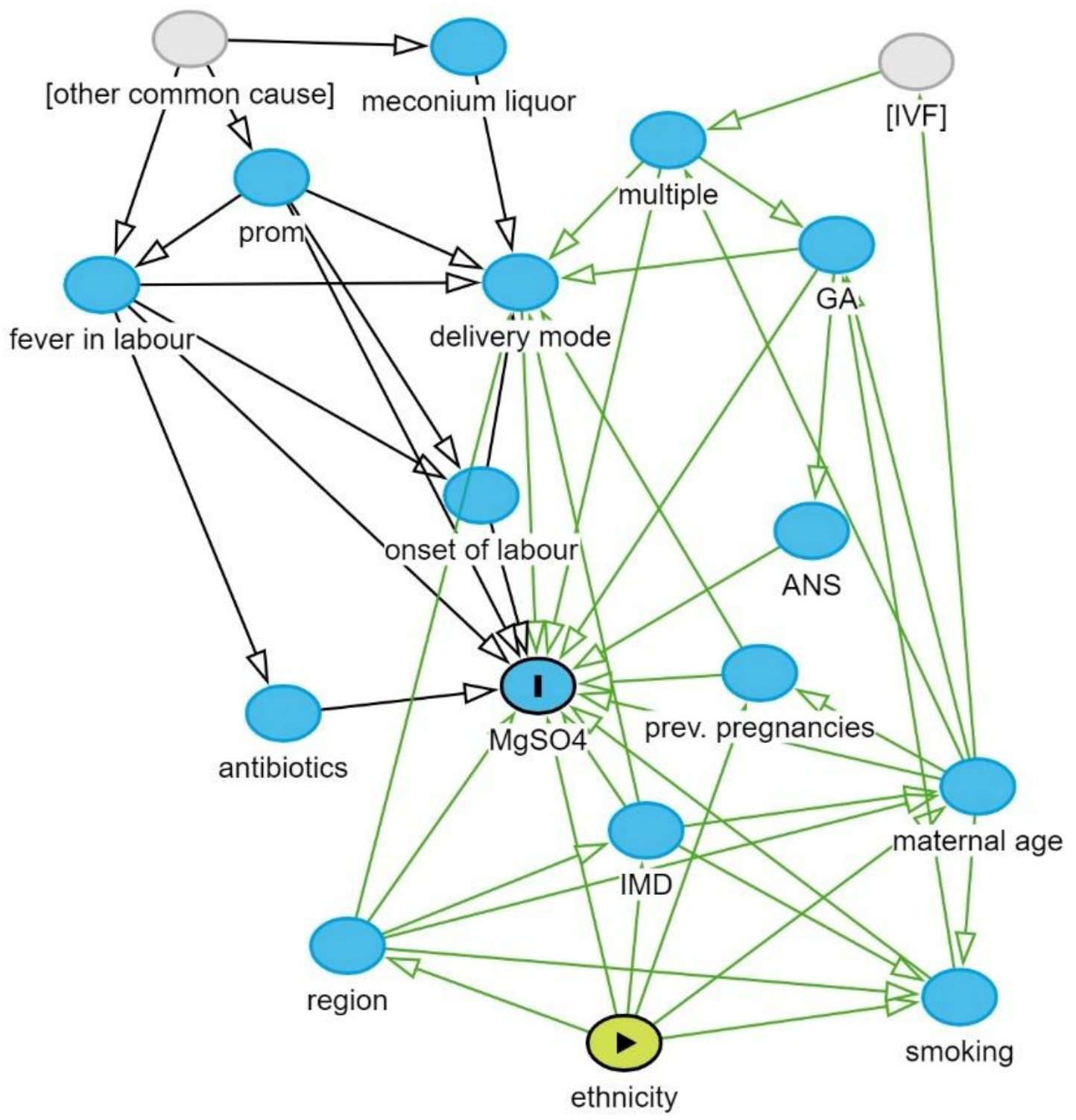

(f) Maternal ethnicity *direct effects* model

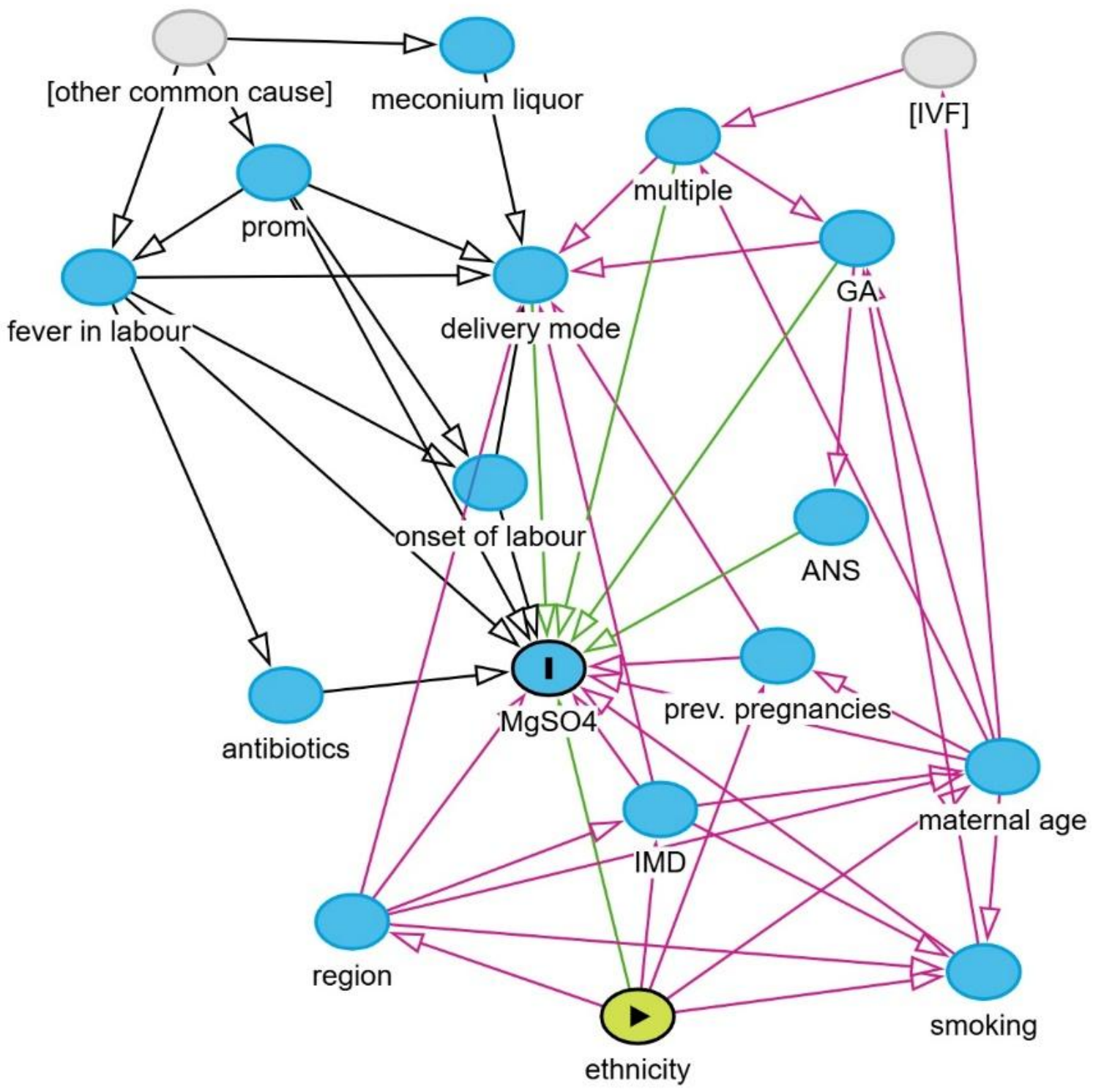

(g) Maternal region *total effects* model

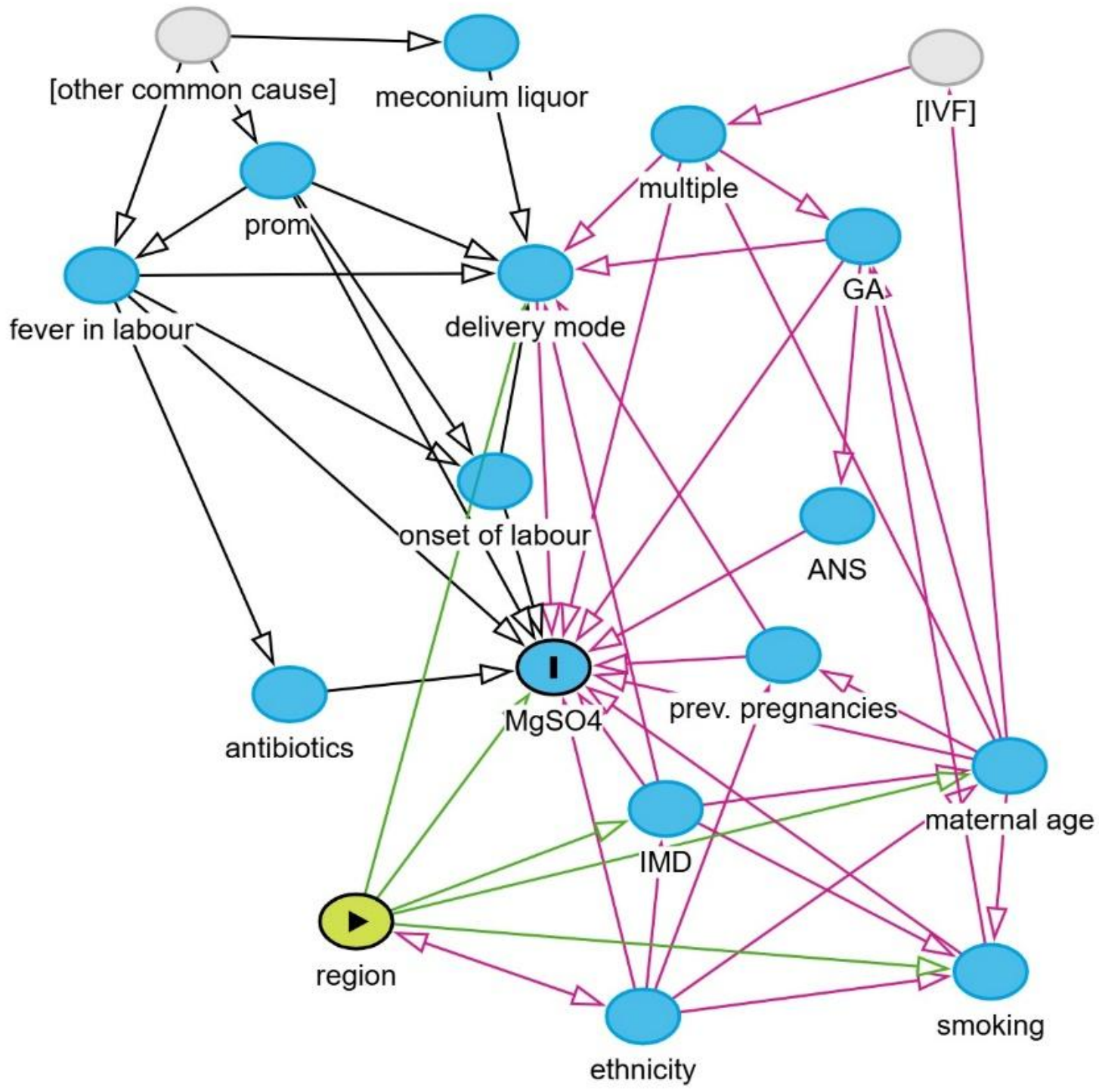

(h) Maternal region *direct effects* model

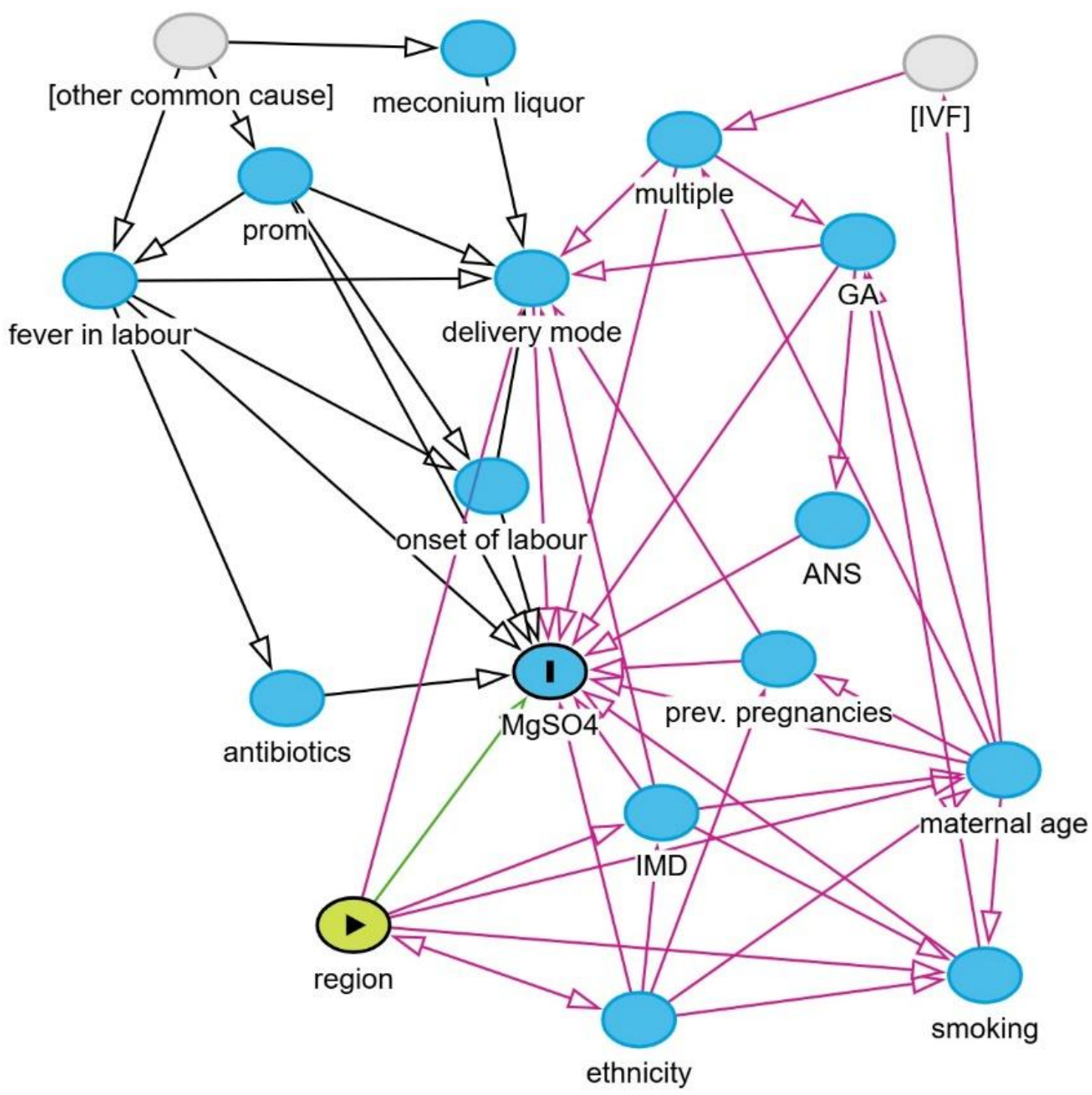

Appendix 3: Directed Acyclic Graphs key

- exposure
- outcome
- ancestor of exposure
- ancestor of outcome
- ancestor of exposure *and* outcome
- adjusted variable
- unobserved (latent)
- other variable
- causal path
- biasing path

Appendix 4: Total and Direct effects of sociodemographic factors on odds of receiving antenatal magnesium sulphate for preterm birth in England 2014-2024

	Unadjusted model[1] (Odds ratio, 95% CI)	Total effects model[1] (Odds ratio, 95% CI)	Direct effects model[1] (Odds ratio, 95% CI)
**Maternal age (≥35 vs <35 years)**			
Overall	1.05 (0.99, 1.11), p=0.127	1.01 (0.94, 1.08), p=0.839	1.04 (0.97, 1.13), p=0.276
p-value for pre/post interaction	p=0.125	p=0.077	p=0.255
Pre-intervention	1.09 (1.01, 1.18), p=0.032	1.06 (0.97, 1.16), p=0.208	1.08 (0.98, 1.19), p=0.120
Post-intervention	0.99 (0.90, 1.09), p=0.828	0.93 (0.84, 1.04), p=0.217	0.99 (0.88, 1.12), p=0.853
**Ethnicity (white vs non-white)**			
Overall	0.88 (0.83, 0.94), p<0.001	0.93 (0.87, 1.00), p=0.038	0.99 (0.91, 1.07), p=0.785
p-value for pre/post interaction	p=0.146	p=0.235	p=0.660
**IMD (more vs less deprived)**			
Overall	0.88 (0.83, 0.93), p<0.001	0.93 (0.86, 0.99), p=0.029	1.00 (0.92, 1.08), p=0.915
p-value for pre/post interaction	p=0.141	p=0.022	p=0.019
Pre-intervention	0.85 (0.79, 0.92), p<0.001	0.87 (0.80, 0.95), p=0.002	0.93 (0.85, 1.03), p=0.152
Post-intervention	0.93 (0.85, 1.02), p=0.133	1.02 (0.92, 1.14), p=0.719	1.11 (0.99, 1.26), p=0.080
**Region (North vs South)**			
Overall	0.73 (0.69, 0.77), p<0.001	0.70 (0.57, 0.85), p<0.001	0.70 (0.57, 0.84), p<0.001
p-value for pre/post interaction	p<0.001	p<0.001	p<0.001
Pre-intervention	0.62 (0.58, 0.66), p<0.001	0.60 (0.49, 0.74), p<0.001	0.60 (0.49, 0.73), p<0.001
Post-intervention	0.96 (0.88, 1.05), p=0.340	0.87 (0.70, 1.08), p=0.201	0.88 (0.71, 1.09), p=0.242

Appendix 5: Figures showing time-trends in MgSO4 uptake by sociodemographic factors

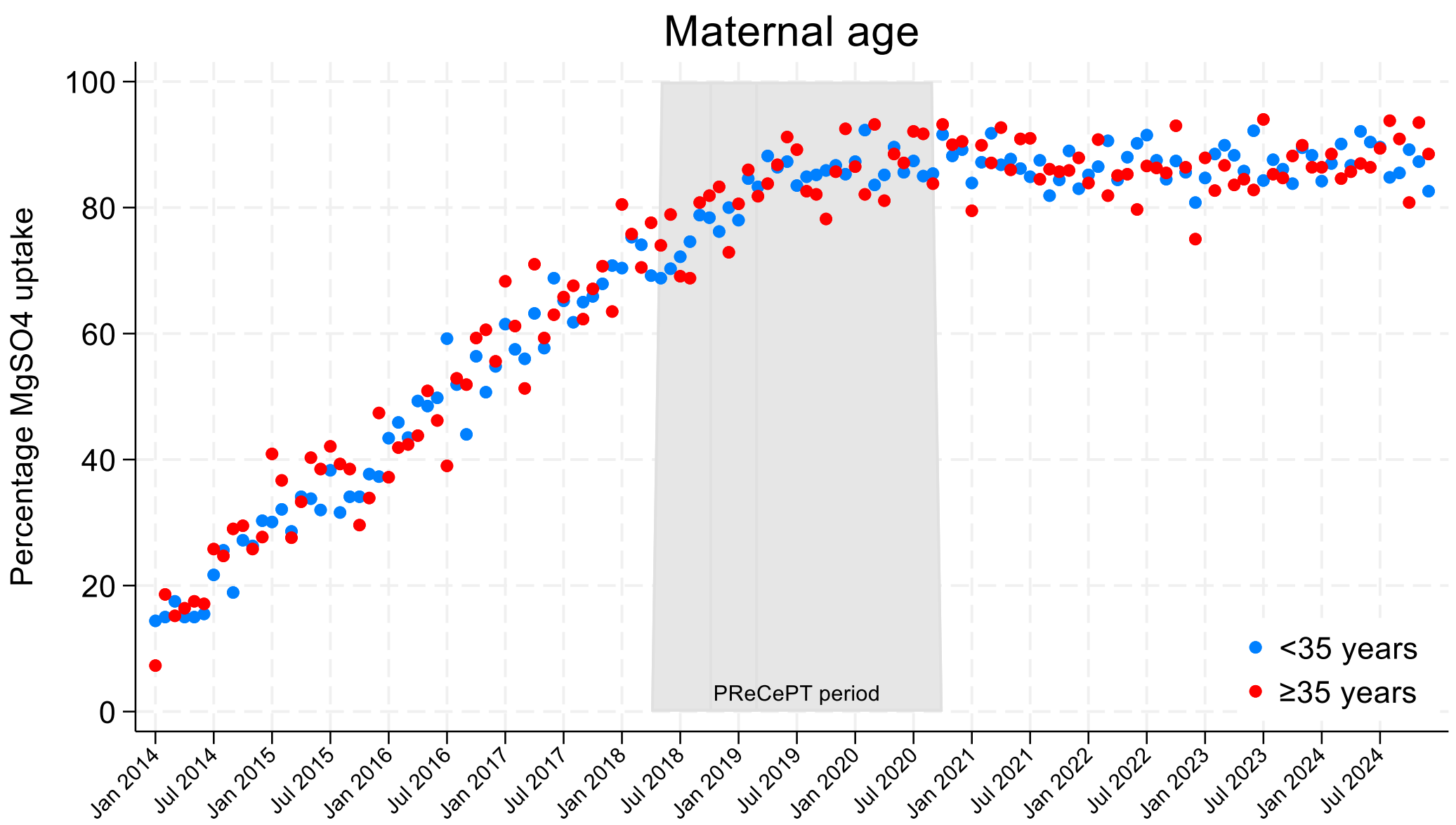

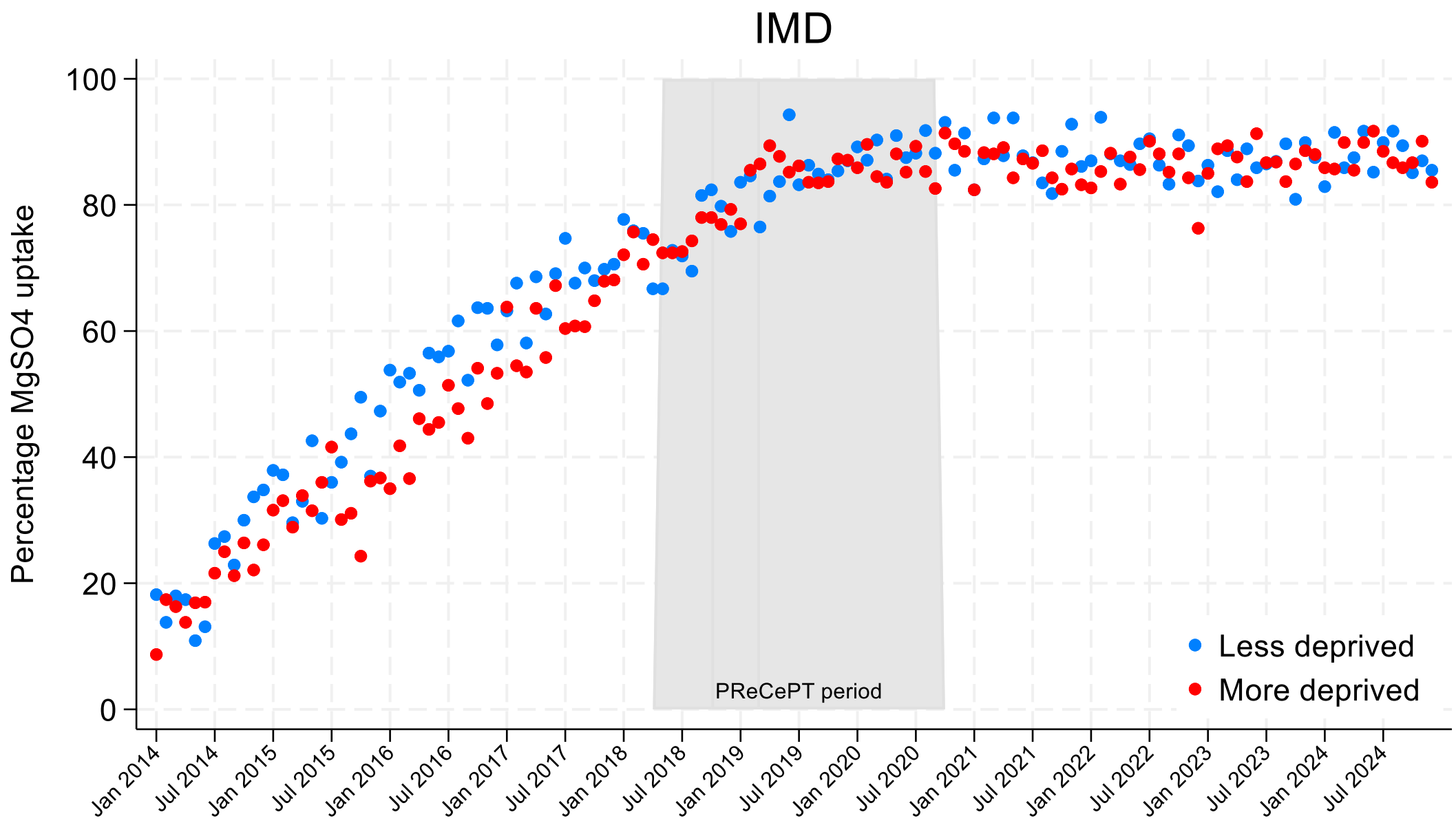

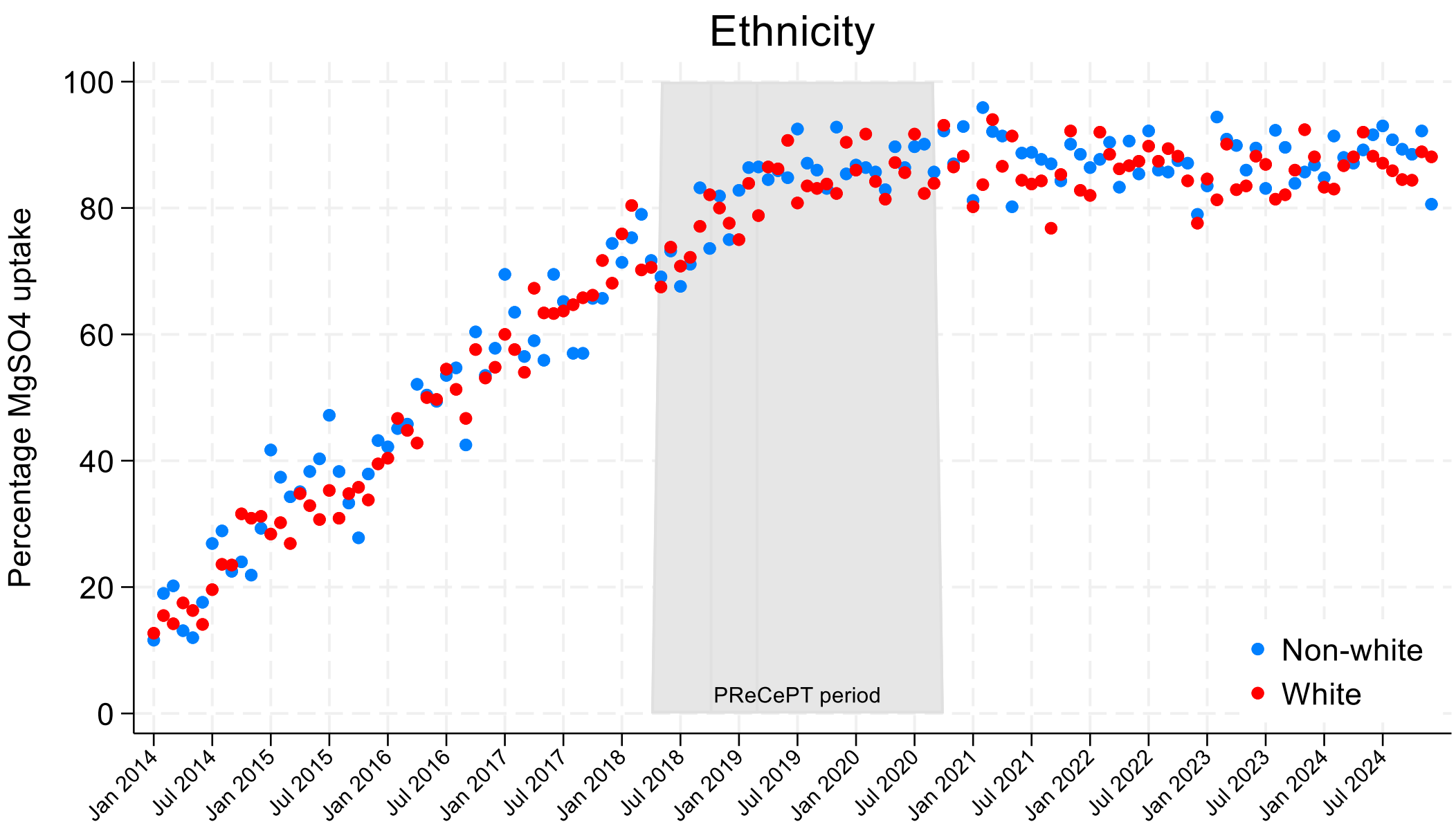
Ethnicity
Percentage MgSO4 uptake
100
80
60
40
20
0
PReCePT period
Non-white
White
Jan 2014
Jul 2014
Jan 2015
Jul 2015
Jan 2016
Jul 2016
Jan 2017
Jul 2017
Jan 2018
Jul 2018
Jan 2019
Jul 2019
Jan 2020
Jul 2020
Jan 2021
Jul 2021
Jan 2022
Jul 2022
Jan 2023
Jul 2023
Jan 2024
Jul 2024

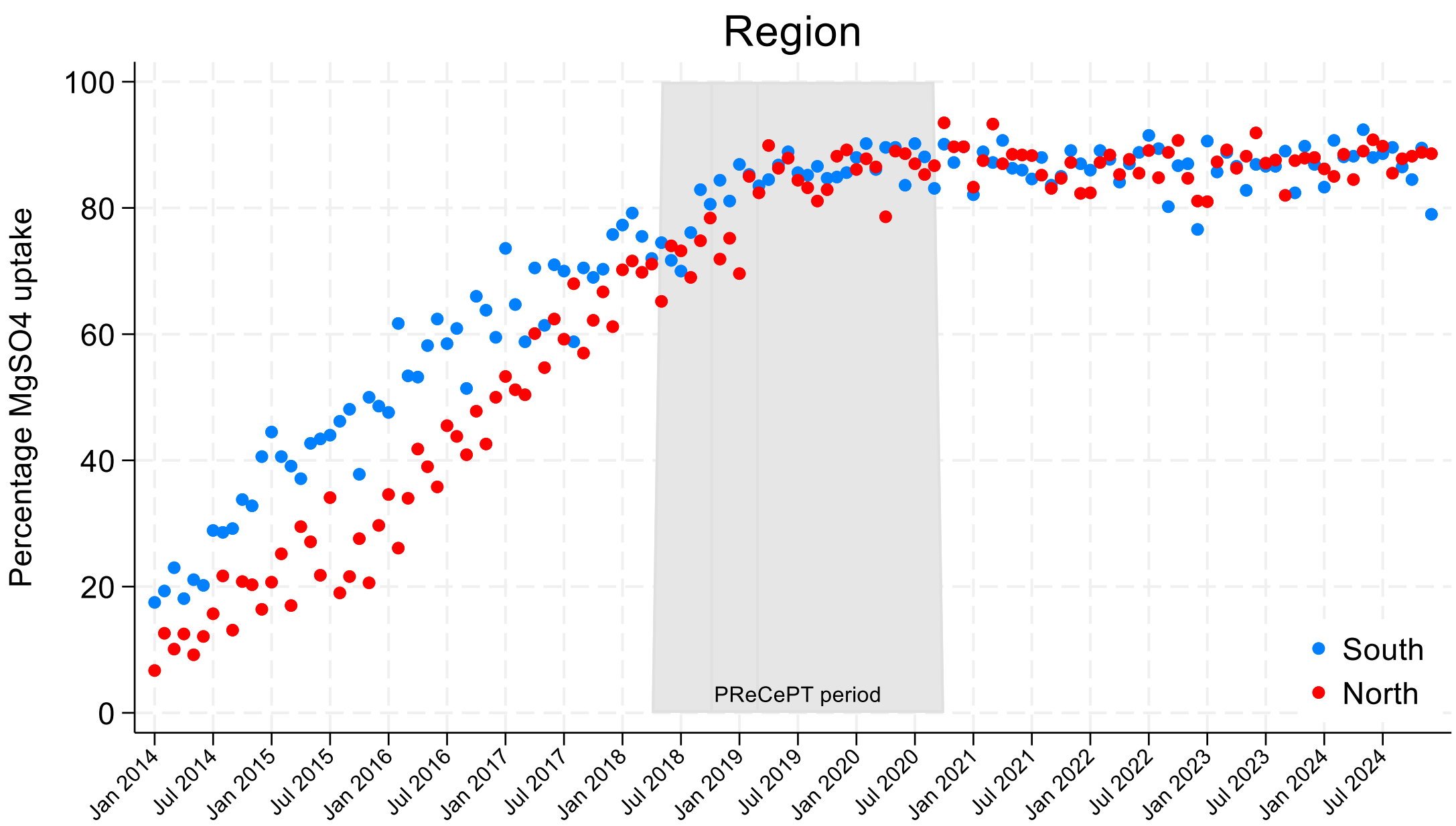
Region
Percentage MgSO4 uptake
100
80
60
40
20
0
PReCePT period
South
North
Jan 2014
Jul 2014
Jan 2015
Jul 2015
Jan 2016
Jul 2016
Jan 2017
Jul 2017
Jan 2018
Jul 2018
Jan 2019
Jul 2019
Jan 2020
Jul 2020
Jan 2021
Jul 2021
Jan 2022
Jul 2022
Jan 2023
Jul 2023
Jan 2024
Jul 2024

Appendix 6: Results of sensitivity analysis excluding mothers with hypertension

	Unadjusted model[1] (Odds ratio, 95% CI)	Total effects model[1] (Odds ratio, 95% CI)	Direct effects model[1] (Odds ratio, 95% CI)
**Maternal age (≥35 vs <35 years)**			
Overall	1.05 (0.99, 1.11), p=0.135	1.00 (0.93, 1.08), p=0.923	1.04 (0.96, 1.12), p=0.349
p-value for pre/post interaction	p=0.126	p=0.084	p=0.281
Pre-intervention	1.09 (1.01, 1.18), p=0.034	1.06 (0.96, 1.16), p=0.245	1.07 (0.97, 1.19), p=0.161
Post-intervention	0.99 (0.90, 1.09), p=0.827	0.93 (0.83, 1.04), p=0.207	0.99 (0.87, 1.11), p=0.823
**Ethnicity (white vs non-white)**			
Overall	0.87 (0.82, 0.93), p<0.001	0.92 (0.86, 0.99), p=0.023	0.98 (0.91, 1.06), p=0.636
p-value for pre/post interaction	p=0.135	p=0.207	p=0.578
**IMD (more vs less deprived)**			
Overall	0.88 (0.83, 0.94), p<0.001	0.93 (0.87, 1.00), p=0.047	1.00 (0.93, 1.09), p=0.911
p-value for pre/post interaction	p=0.148	p=0.022	p=0.022
Pre-intervention	0.85 (0.79, 0.92), p<0.001	0.87 (0.80, 0.96), p=0.003	0.94 (0.85, 1.04), p=0.217
Post-intervention	0.93 (0.85, 1.02), p=0.144	1.03 (0.92, 1.15), p=0.642	1.12 (0.99, 1.27), p=0.067
**Region (North vs South)**			
Overall	0.72 (0.68, 0.76), p<0.001	0.69 (0.56, 0.84), p<0.001	0.69 (0.56, 0.84), p<0.001
p-value for pre/post interaction	p<0.001	p<0.001	p<0.001
Pre-intervention	0.60 (0.56, 0.65), p<0.001	0.59 (0.48, 0.73), p<0.001	0.58 (0.48, 0.72), p<0.001
Post-intervention	0.96 (0.88, 1.04), p=0.316	0.87 (0.70, 1.08), p=0.206	0.89 (0.71, 1.10), p=0.270

Appendix 7: Figures from sensitivity analysis excluding mothers with pregnancy hypertension

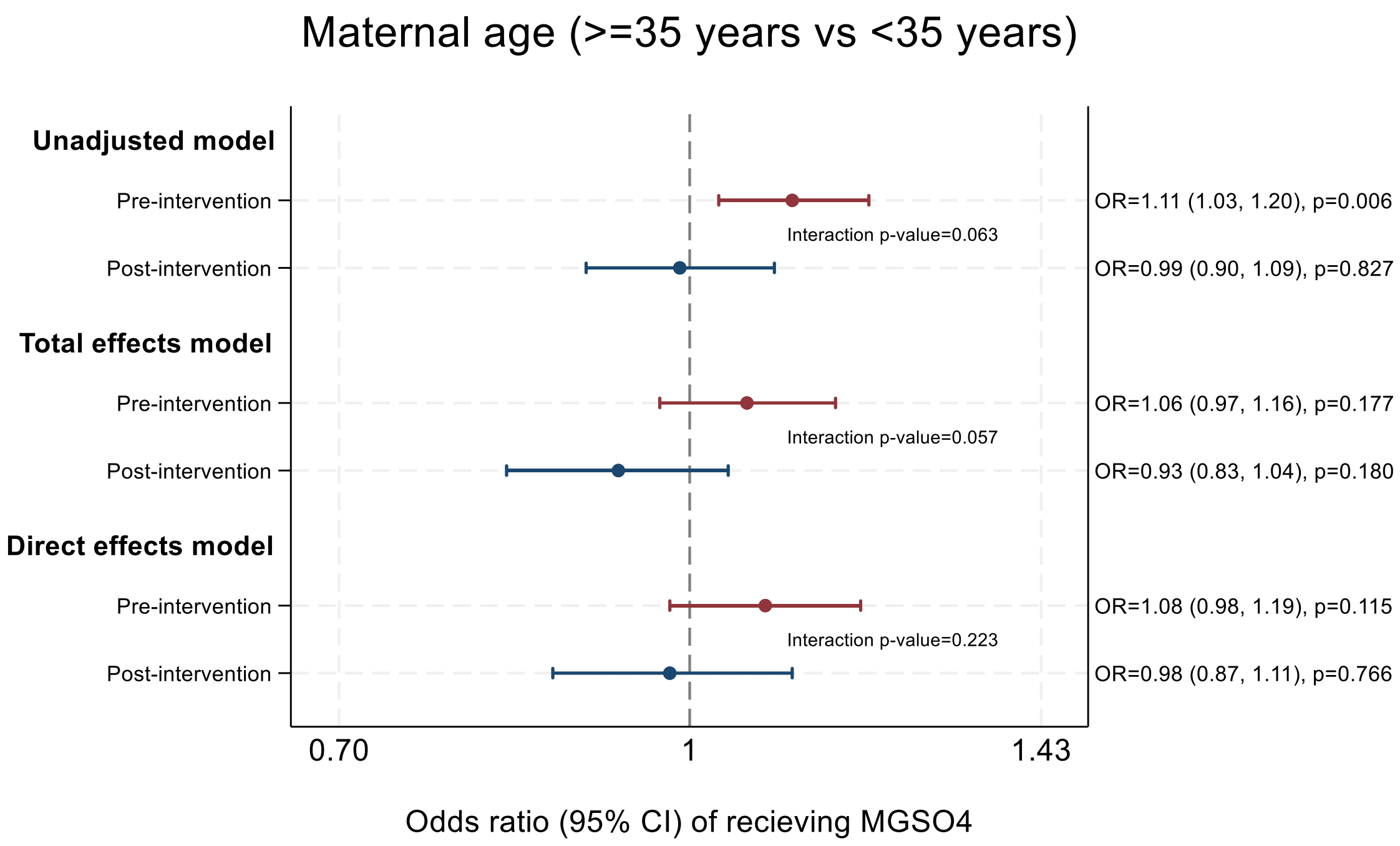

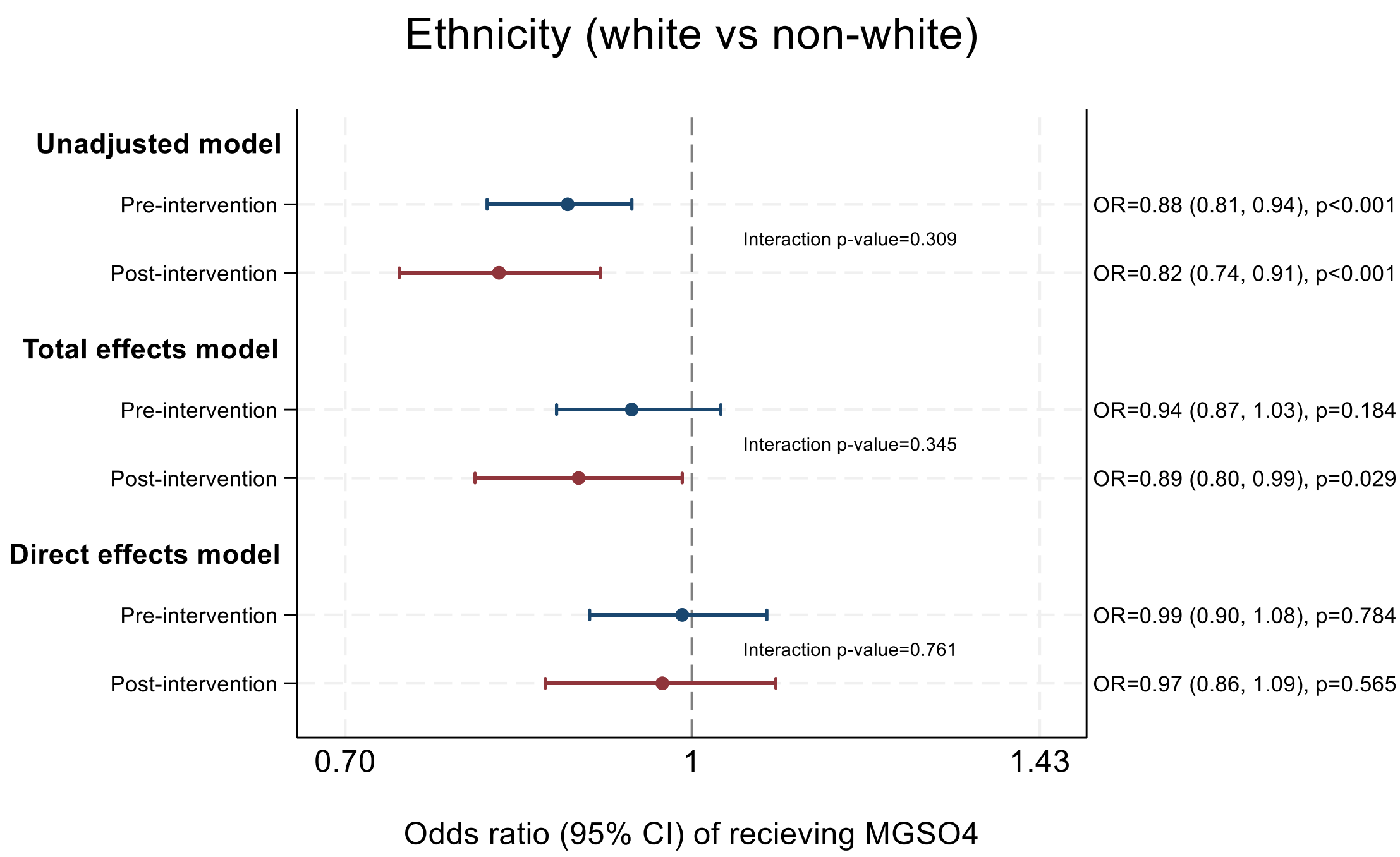

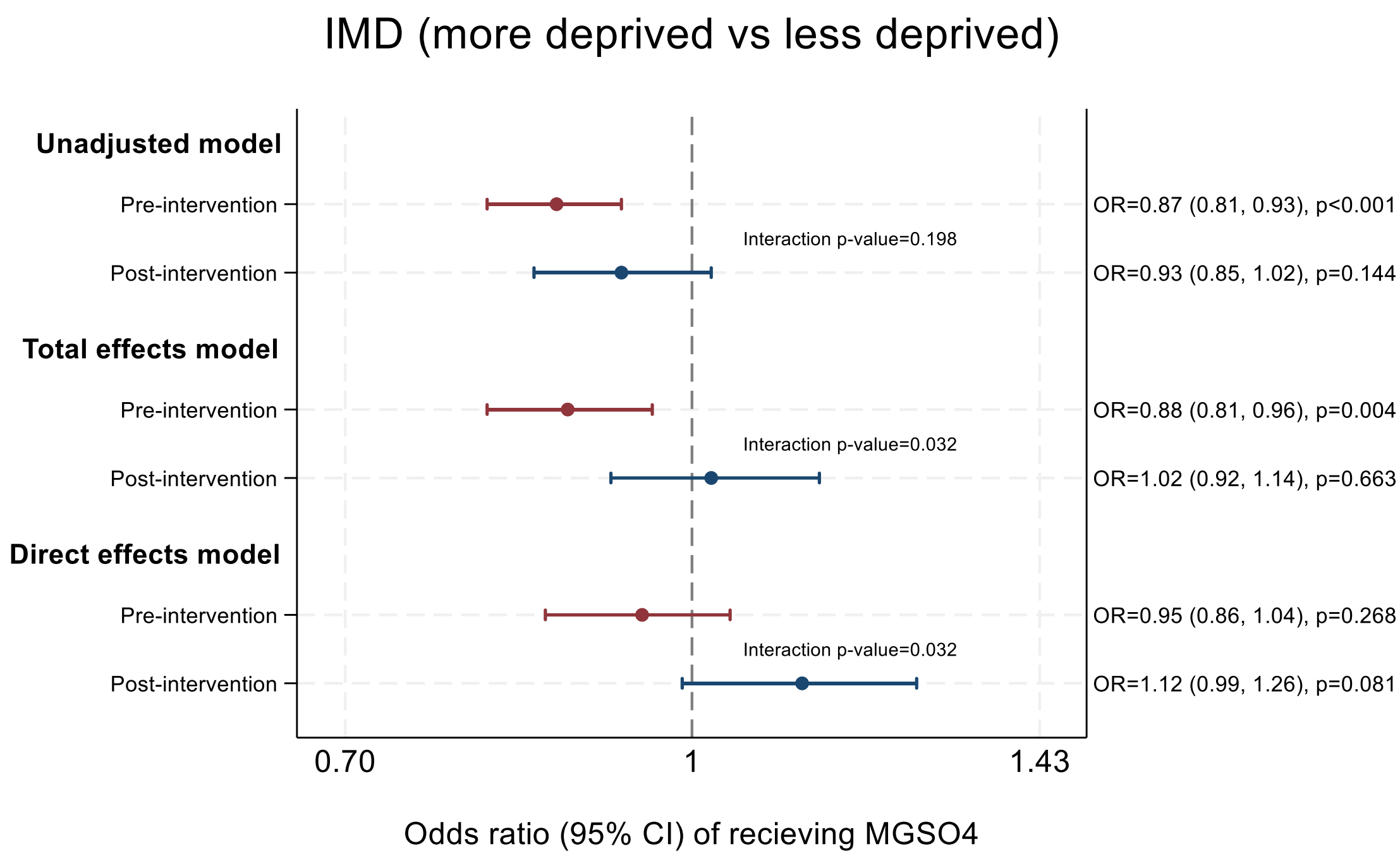
IMD (more deprived vs less deprived)
Unadjusted model
Pre-intervention
Post-intervention
Interaction p-value=0.198
OR=0.87 (0.81, 0.93), p<0.001
OR=0.93 (0.85, 1.02), p=0.144
Total effects model
Pre-intervention
Post-intervention
Interaction p-value=0.032
OR=0.88 (0.81, 0.96), p=0.004
OR=1.02 (0.92, 1.14), p=0.663
Direct effects model
Pre-intervention
Post-intervention
Interaction p-value=0.032
OR=0.95 (0.86, 1.04), p=0.268
OR=1.12 (0.99, 1.26), p=0.081
0.70
1
1.43
Odds ratio (95% CI) of recieving MGSO4

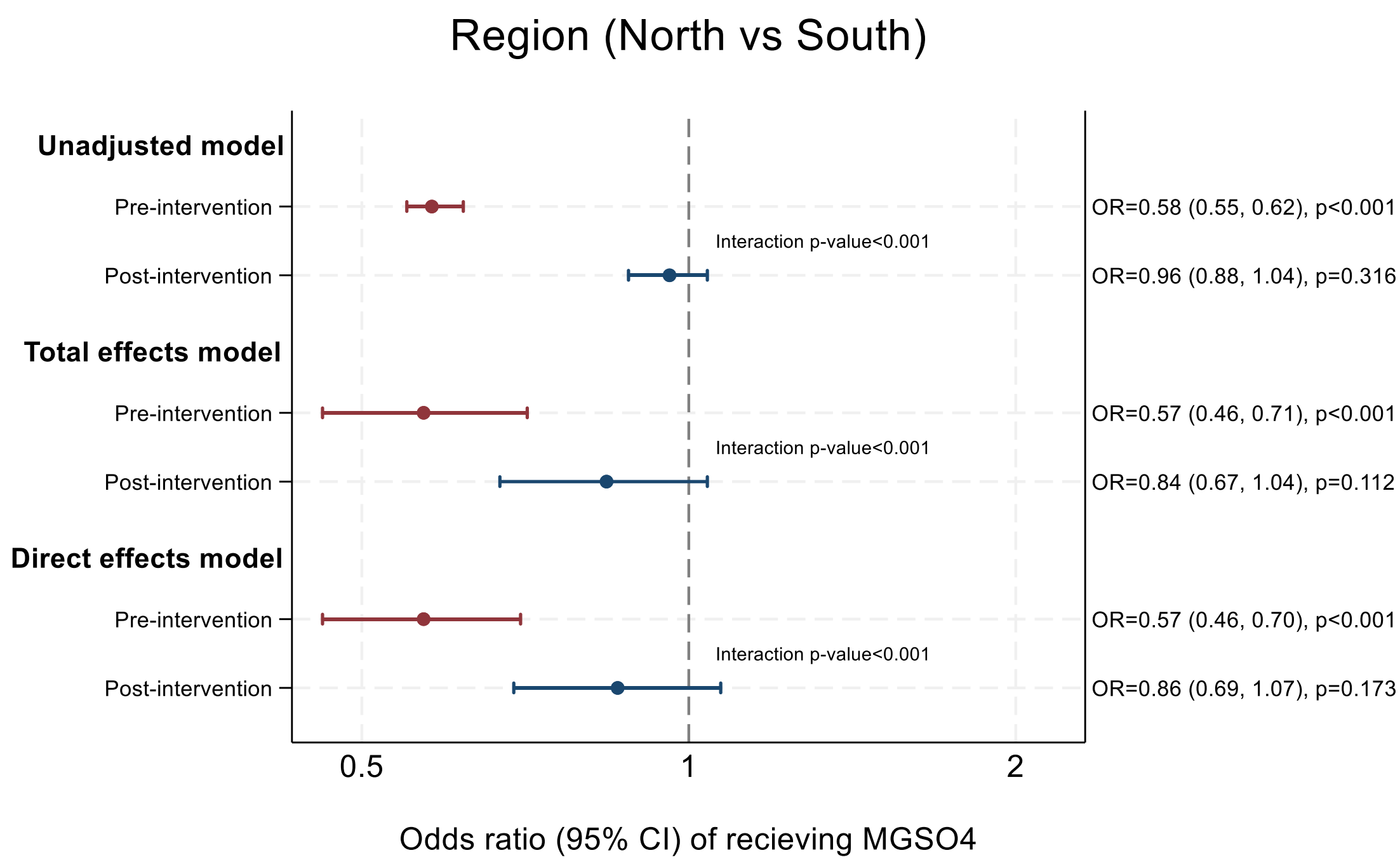
Region (North vs South)
Unadjusted model
Pre-intervention
Post-intervention
Interaction p-value<0.001
OR=0.58 (0.55, 0.62), p<0.001
OR=0.96 (0.88, 1.04), p=0.316
Total effects model
Pre-intervention
Post-intervention
Interaction p-value<0.001
OR=0.57 (0.46, 0.71), p<0.001
OR=0.84 (0.67, 1.04), p=0.112
Direct effects model
Pre-intervention
Post-intervention
Interaction p-value<0.001
OR=0.57 (0.46, 0.70), p<0.001
OR=0.86 (0.69, 1.07), p=0.173
0.5
1
2
Odds ratio (95% CI) of recieving MGSO4
